# Rhinacanthin C Alleviates Amyloid-*β* Fibrils' Toxicity on Neurons and Attenuates Neuroinflammation Triggered by LPS, Amyloid-*β*, and Interferon-*γ* in Glial Cells

**DOI:** 10.1155/2017/5414297

**Published:** 2017-10-18

**Authors:** Kai-An Chuang, Ming-Han Li, Ni-Hsuan Lin, Chih-Hsuan Chang, I-Huang Lu, I-Hong Pan, Tomoya Takahashi, Ming-Der Perng, Shu-Fang Wen

**Affiliations:** ^1^Biomedical Technology and Device Research Laboratories, Industrial Technology Research Institute, 321 Kuang Fu 2nd Road, Hsinchu 30011, Taiwan; ^2^Institute of Molecular Medicine, College of Life Sciences, National Tsing Hua University, 101 Kuang Fu 2nd Road, Hsinchu 30013, Taiwan; ^3^ARSOA Research & Development Center, AOB Keioh Group Corp., 2961 Kobuchisawa-cho, Hokuto, Yamanashi 408-8522, Japan

## Abstract

Neuroinflammation plays a central role in the pathophysiology of Alzheimer's disease (AD). Compounds that suppress neuroinflammation have been identified as potential therapeutic targets for AD. Rhinacanthin C (RC), a naphthoquinone ester found in *Rhinacanthus nasutus* Kurz (Acanthaceae), is currently proposed as an effective molecule against inflammation. However, the exact role of RC on neuroinflammation remains to be elucidated. In the present study, we investigated RC effect on modulating lipopolysaccharides (LPS), amyloid-*β* peptide (A*β*), or interferon-*γ*- (IFN-*γ*-) evoked pathological events in neurons and glia. Our findings demonstrated that RC prevented A*β*-induced toxicity in rat hippocampal neurons and attenuated LPS-activated nitric oxide (NO) production, inducible nitric oxide synthase (iNOS) expression, and NF-*κ*B signaling in rat glia. Likewise, RC suppressed LPS-induced neuroinflammation by reducing NO production and iNOS, IL-1*β*, CCL-2, and CCL-5 mRNA levels in rat microglia. Further studies using BV-2 microglia revealed that RC inhibited LPS-, A*β*-, and IFN-*γ*-stimulated IL-6 and TNF-*α* secretion. Of note, NF-*κ*B and ERK activation was abrogated by RC in BV-2 cell response to A*β* or IFN-*γ*. Moreover, RC protected neurons from A*β*-stimulated microglial conditioned media-dependent toxicity. Collectively, these data highlight the beneficial effects of RC on neuroprotection and support the therapeutic implications of RC to neuroinflammation-mediated conditions.

## 1. Introduction

Neuroinflammation is an inflammatory reaction within the central nervous system (CNS) and currently one of the major components contributing to Alzheimer's disease (AD) pathology [1]. The features of neuroinflammation include infiltration of immune cells, activation of glial cells, and production of inflammatory mediators [2]. Microglia, the resident phagocytes of the CNS, represent approximately 5–12% of the brain population [3]. Like most tissue macrophages, microglia patrol the brain for pathogens and support CNS homeostasis and plasticity with constant motility [4]. Upon sensing damage, such as protein aggregates, microglia respond by adopting a set of morphological and functional attributes, that is, “reactive” or “primed” [5]. While microglia's response to injury is typically beneficial, it can go awry, particularly in cases of chronic injury and long-term inflammation, such as in neurodegenerative diseases [6]. Due to the neuronal damage in AD cannot be completely explained by the “amyloid cascade hypothesis” [7], more attention has been paid to the potential role of microglia in disease etiology, buoyed by the compelling genetic associations between microglial pathways and AD [8, 9].

Controlling the activation of microglia has become a major target in AD and other neurodegenerative diseases of aging [10]. Highly activated microglia have been seen in Tg mouse models of AD and in old normal mice [11, 12]. Further evidence comes from the presence of microglia surrounding amyloid plaques in AD cerebral cortex and the presence of A*β* deposits in T-cells, activated microglia, and reactive astrocytes in the brains of AD patients [13, 14]. Activated microglia release proinflammatory and cytotoxic factors, including TNF-*α*, IL-1*β*, IL-6, nitric oxide (NO), and reactive oxygen species (ROS) [15]. These mediators may directly induce neuronal apoptosis or amplify the local inflammatory response, which leads to possible synaptic dysfunction or neuronal loss [16]. Neuronal damage or death may also induce microglial activation, which facilitates the propagation of a localized, detrimental cycle of neuroinflammation [17]. Apart from a direct neurotoxic effect, activated microglia can promote A*β* deposition under inflammatory conditions as studied in mouse models and in the brains of AD patients [18, 19]. In fact, microglia have the ability to phagocytose stressed or dying neurons and express phagocytic receptors on their surface [20]. Inflammatory stimuli found to activate microglial phagocytosis of neurons include lipopolysaccharide (LPS) and IFN-*γ* that are able to produce a massive inflammatory response characterized by producing and secreting IL-6, TNF-*α*, IL-1*β*, IL-12, and iNOS, all of which can confer neurotoxicity [21, 22]. Additionally, A*β* fibrils can also induce inflammatory processes by binding to and activating microglia [23].

Rhinacanthin C (RC) is a naphthoquinone ester found in *Rhinacanthus nasutus* (L.) Kurz (Acanthaceae) [24] that is widely distributed in Southeast Asia for the treatment of diabetes, hypertension, hepatitis, pneumonia, pulmonary tuberculosis, cancer, and skin diseases [25–28]. Recent studies have shown that *Rhinacanthus nasutus* exhibited anti-inflammatory [29] and neuroprotective activity against hypoxia-, glutamate-, or A*β*-induced cell death [30, 31]. Specifically, lupeol, stigmasterol, and *β*-sitosterol are identified as the molecules against glutamate toxicity. Likewise, RC has also been reported to exert various biological functions, including antihyperglycaemia, antihyperlipidemia [32], anti-inflammatory [33, 34], antiosteoclastogenesic [35], antiallergic [36], antitumor [37], antimicrobial [24, 38, 39], and antimosquito [40] activities. With respect to anti-inflammatory activity, RC was able to diminish NO and prostaglandin E2 release in LPS-stimulated RAW264.7 macrophages [33] and inhibits abnormal bone lysis that occurs during inflammatory bone resorption [35]. More recently, Chang and colleagues have demonstrated that RC possesses neuroprotective effect via modulating high-mobility group box 1-related neuroinflammation and attenuating brain apoptosis in a rat subarachnoid hemorrhage model [34]. Although being implicated in regulation of various inflammatory actions, the exact role of RC in neuroinflammation remains to be elucidated. The aim of this study is to investigate whether RC can modulate glial functions as part of an attempt to understand the possible immunomodulatory properties of this compound and its functioning mechanisms against neuroinflammation.

## 2. Materials and Methods

### 2.1. Cell Culture

All experiments involving animals were approved by the Institutional Animal Care and Use Committee of the National Tsing Hua University (NTHU-0010327). Primary hippocampal neurons were prepared from fetal brain of Sprague Dawley rats (BioLASCO Taiwan, Taipei, Taiwan) at embryonic day 18 (E18) as described previously [41] with slight modifications. In brief, hippocampi were dissected in Hank's balanced salt solution (HBSS) followed by incubation with papain (10 U/mL) at 37°C for 10 min. After incubating with DNase I (Sigma-Aldrich, St. Louis, MO, USA) at 37°C for additional 10 min, tissues were mechanically dispersed by triturating with a Pasteur pipette. Cells were collected by centrifugation at 1000 rpm for 5 min and resuspended in plating medium (MEM containing 10% (*v/v*) horse serum, 100 U/mL penicillin, and 100 *μ*g/mL streptomycin). After filtration through a 70 micron nylon mesh (Greiner Bio-One, Frickenhausen, Germany), cells were seeded onto poly-L-lysine-coated plates or dishes at a density of 2.5 × 10^4^ cells/cm^2^. After 12–16 h, the plating medium was replaced with neurobasal medium containing 2% (*v/v*) B27, 0.5 mM glutamine, 100 U/mL penicillin, and 100 *μ*g/mL streptomycin (all from Thermo Fisher Scientific, Waltham, MA, USA). Cells were allowed to mature *in vitro* for at least 8 days prior to treatment, and half of the medium was replaced with fresh neurobasal medium every 3-4 days. The purity of these cultures was assessed by immunostaining with specific markers for neurons (*β*-III tubulin) and astrocytes (GFAP), with >95% positive for *β*-III tubulin.

*Primary cortical glial cultures* were prepared from the fetal brain of Sprague Dawley rats at embryonic day 18 (E18). The cortices were dissected in HBSS followed by incubation with 0.25% (*w/v*) trypsin at 37°C for 15 min. After incubation with DNase I (Sigma-Aldrich) for an additional 5 min, the cortices were mechanically dispersed by triturating with a Pasteur pipette. Dissociated cortical cells were collected by centrifugation at 1000 rpm for 5 min and resuspended in plating medium (MEM supplemented with 5% (*v/v*) horse serum, 5% (*v/v*) fetal bovine serum (FBS), 100 U/mL penicillin, and 100 *μ*g/mL streptomycin). Unless otherwise stated, all cell culture reagents were purchased from Thermo Fisher Scientific. After filtration through a 40 *μ*m nylon mesh (Greiner Bio-One), cells were seeded onto poly-L-lysine-coated flasks at 5 × 10^4^ cells/cm^2^. Cells were maintained in a humidified 5% CO_2_ atmosphere at 37°C, and medium was changed every 3-4 days. Experiments were performed at 8–10 days *in vitro*. These cultures contain 95% astrocytes as determined by immunofluorescence microscopy using a monoclonal antibody specific to GFAP.

Primary microglia were prepared from mixed cortical astroglial/microglial cultures by shaking the flasks at 200 rpm for 2 h at 37°C. After centrifugation at 2000 ×g for 5 min, the resulting cell pellet was resuspended in N2 medium (50% (*v/v*) neurobasal, 50% (*v/v*) MEM, 1% (*v/v*) N2 supplement, 100 U/mL penicillin, 100 *μ*g/mL streptomycin, and 2 mM glutamine) and plated onto 60 mm dishes, 6-well plates, 12-well plates, or 96-well plates as needed. The purity of these cultures is typically >97% microglia as determined by immunostaining using an anti-Iba-1 antibody (Wako Chemicals, Richmond, VA, USA).

The BV-2 mouse microglial cell line was a kind gift from Dr. Chiou-Feng Lin (Department of Microbiology and Immunology, Taipei Medical University, Taiwan). The cells were maintained in RPMI-1640 medium supplemented with 10% FBS, 100 U/mL penicillin, and 100 *μ*g/mL streptomycin (all from Thermo Fisher Scientific) at 37°C, 5% CO_2_, and plated at a density of 2.5 × 10^4^ cells/cm^2^ onto 6-well plates, 12-well plates, or 96-well plates as needed.

### 2.2. Preparation of Rhinacanthin C

Rhinacanthin C (RC) was isolated from *Rhinacanthus nasutus* that was provided by the AOB KEIOH GROUP Corp. The roots (2 kg) of *Rhinacanthus nasutus* were extracted with 90% ethanol three times under reflux for 1 h. The 90% ethanol extract (77.82 g) was then partitioned with 90% methanol and *n*-hexane. The 90% methanol layer was concentrated to afford a brown syrup (54.32 g) and further partitioned between CH_2_Cl_2_ and water. The CH_2_Cl_2_ layer was concentrated to give a brown syrup (27.55 g), 2.50 g of which was chromatographed on a silica gel column eluted with *n*-hexane/EtOAc (*v/v*, 4 : 1) to obtain RC (444.2 mg) after concentration. RC was dissolved in dimethyl sulfoxide (DMSO) to a concentration of 10 mM, and aliquots were stored at −20°C.

### 2.3. Cell Treatments

In all experiments, cells were allowed to adhere for 24 h and then stimulated with A*β*, LPS, or IFN-*γ* for 24 h or 48 h unless otherwise stated. Where indicated, RC was added 1–4 h beforehand.

### 2.4. Preparation of A*β* Fibrils

A*β*_25–35_ (Gly-Ser-Asn-Lys-Gly-Ala-Ile-Ile-Gly-Leu-Met) was purchased from Sigma-Aldrich. One milligram of A*β* peptide was dissolved in 1 mL of distilled water to a concentration of 1 mg/mL. To prepare A*β* fibrils, the solution was incubated at 37°C for 7 days. Aliquots of the stock solutions were stored at −20°C. Fibril formation was examined using transmission electron microscopy.

### 2.5. Electron Microscopy

A*β* fibrils spread on carbon-coated copper grids (Ted Pella Inc., Redding, CA, USA) were negatively stained with 1% (*w/v*) uranyl acetate (Electron Microscopy Sciences, Hatfield, PA, USA) and examined with an HT7700 transmission electron microscope (HT7700, Hitachi High-Technologies, Tokyo, Japan) with an accelerating voltage of 100 kV. Images were acquired using a CCD (charge-coupled device) camera before being further processed in Adobe Photoshop CS6 (Adobe, San Jose, CA, USA).

### 2.6. Phase-Contrast Microscopy

Primary rat neurons on poly-L-lysine were treated with 25 *μ*M A*β* for 24 h. Images were acquired with the Zeiss Axiovert 40C light microscope (Carl Zeiss, Jena, Germany) with 20x objective lens.

### 2.7. Immunofluorescence Microscopy

For double-label immunofluorescence microscopy, cells were fixed in 4% (*w/v*) paraformaldehyde (PFA) (Electron Microscopy Sciences, Hatfield, PA, USA) in phosphate-buffered saline (PBS) for 10 min and permeabilized with 0.2% (*v/v*) Triton X-100 in PBS for 5 min at room temperature. Unless otherwise stated, all of the following steps were conducted at room temperature. After a 5-minute wash with 0.1% (*w/v*) bovine serum albumin (BSA) (Sigma-Aldrich, St. Louis, MO, USA) in PBS, cells were blocked with 10% (*w/v*) normal goat serum (NGS) (Jackson ImmunoResearch Laboratories, West Grove, PA, USA) in PBS for 20 min. After being washed with PBS, cells were incubated with primary antibodies for 1 h. The primary antibodies used in this study were anti-*β*-III tubulin (1 : 400; BioLegend, San Diego, CA, USA), cleaved caspase-3 (1 : 200; Cell Signaling Technology, Beverly, MA, USA), and caspase-6-generated tubulin antibody (1 : 500; tubulinΔCsp6), kindly provided by Professor Andréa C. LeBlanc (The Bloomfield Center for Research in Aging, Lady Davis Institute for Medical Research, Jewish General Hospital, Canada) [42]. Cells were washed three times with 0.1% (*w/v*) BSA in PBS, followed by incubation for 1 h with Alexa Fluor® 488 (1 : 500) or Alexa Fluor 594-conjugated (1 : 500) goat anti-mouse, anti-rabbit, or anti-rat secondary antibodies (Thermo Fisher Scientific). All antibodies were diluted in PBS containing 1% (*w/v*) NGS. In some experiments, cells were counterstained with 25 ng/mL of 4′,6-diamidino-2-phenylindole (DAPI; Thermo Fisher Scientific) to visualize nuclei. After mounting on glass slides using ReadiUse™ microscope mounting solution (AAT Bioquest, Sunnyvale, CA, USA), the coverslips were observed using a LSM 510 confocal laser scanning microscope (Carl Zeiss, Jena, Germany) with a 40x (0.75 numerical aperture [NA]) Plan-Neofluar Apochromat objective lens. Images were collected in Multitrack mode by LSM 510 software taking 1.0 *μ*m optical sections and processed for images using Photoshop CS6 (Adobe, San Jose, CA, USA).

### 2.8. Cell Fractionation and Immunoblotting

Primary rat hippocampal neurons grown on dishes were washed with PBS and lysed using radioimmunoprecipitation assay (RIPA) lysis buffer (1% (*v/v*) Triton X-100, 0.5% (*w/v*) sodium deoxycholate, 0.1% (*w/v*) SDS, 150 mM NaCl, 50 mM Tris-HCl, pH 8, 2 mM EGTA) supplemented with 1 mM phenylmethylsulfonyl fluoride (PMSF), a cocktail of protease inhibitors (Roche, Mannheim, Germany), and phosphatase inhibitors (Merck Millipore, Billerica, MA, USA). Cell lysates were then homogenized on ice in a 1 mL Dounce homogenizer (Wheaton, Millville, NJ, USA). After determination of protein concentration, total cell lysates were centrifuged at 16,000 ×g for 10 min at 4°C in a benchtop centrifuge (Eppendorf AG, Hamburg, Germany). The resulting supernatants were collected as soluble fractions, and the remaining pellets, representing the insoluble fractions, were resuspended in Laemmli's sample buffer [43] in a volume that was equivalent to the supernatant. In some experiments of primary rat glial cells, whole cell lysates were collected in RIPA buffer. Where indicated, the glial cells were lysed using the Nuclear/Cytosol Extraction Kit (BioVision, Milpitas, CA) and the cytosol and nuclear fractions were separated according to the manufacturer's protocol.

Immunoblotting was performed using the wet electrophoretic transfer system (Bio-Rad, Hercules, CA, USA) according to the manufacturer's instructions. The membranes were washed with Tris-buffered saline (TBS, 150 mM NaCl, 20 mM Tris-HCl, pH 7.4) three times, followed by incubation in the blocking buffer (3% (*w/v*) BSA in TBS containing 0.1% (*v/v*) Tween 20) for 1 h at room temperature. The primary antibodies used were as follows: *β*-III tubulin (1 : 5000; BioLegend), cleaved caspase-3 (1 : 5000; Cell Signaling Technology), tubulinΔCsp6 (1 : 5000; from Professor Andréa C. LeBlanc) [42], iNOS (1 : 5000; Novus Biologicals, Littleton, CO, USA), NF-*κ*B p65 (1 : 5000; Cell Signaling Technology), lamin A/C (1 : 5000; Cell Signaling Technology), and *β*-actin (1 : 5000; Novus Biologicals). After being washed with TBS containing 0.1% (*v/v*) Tween 20, membranes were incubated with horseradish peroxidase- (HRP-) conjugated goat anti-mouse, anti-rabbit, or anti-rat secondary antibodies (Jackson ImmunoResearch Laboratories) for 2 h at room temperature. Peroxidase activity was revealed using an enhanced chemiluminescence (ECL) detection kit (Thermo Fisher Scientific) and visualized using a LAS 4000 (GE Healthcare, Chicago, IL, USA) imaging system. The strength of signals was quantified using the ImageQuant TL 7.0 software (IQTL ver 7.0, GE Healthcare) and adjusted for protein loading by normalizing to *β*-actin or lamin A/C.

### 2.9. Neurite Outgrowth Assay

Primary rat hippocampal cultures were prepared from Sprague Dawley rat embryos and differentiated for 10 days at 37°C in a humidified 5% CO_2_ incubator before treatment. To investigate the effect of RC on neurite outgrowth, 1 h after the treatment of RC, 5 *μ*M A*β*_25–35_ was added to the cell culture for 24 h. Cells were subjected to fluorescence immunostaining. After fixation with 4% PFA, cells were permeabilized with Triton X-100 and incubated with *β*-III tubulin antibody (BioLegend) for 1 h at room temperature, followed by incubation with Alexa Fluor 488-conjugated goat anti-mouse secondary antibody (Thermo Fisher Scientific). Cells were mounted on coverslips and observed under a confocal laser microscope. Three microscopic fields per well per condition were quantified. The *β*-III tubulin area was quantified using ImageJ as described previously [44]. The total neurite length was determined using NeuronJ [45], and the scale bar was used as a size reference.

### 2.10. Treatment of Neurons with Microglial Conditioned Media

Microglia seeded onto poly-L-lysine-coated 12-well plates at a density of 1 × 10^5^ cells/well were treated with 5 *μ*M A*β* in the absence or presence of RC for 24 h. When stated, cells were pretreated for 1 h with 0.5 *μ*M RC. Microglial conditioned media were collected, centrifuged at 2000 ×g for 5 min, and filtered through 0.2 *μ*m syringe filters (Pall Corporation, Hamburg, NY, USA). Primary hippocampal neurons cultured for 14 days were treated with 50% microglial conditioned media for 24 h before staining with anti-Tau antibody (1 : 500, Cell Signaling Technology) to specifically label axons for immunofluorescence microscopy analysis. Tau-positive area was quantified by ImageJ, and the axon length was measured using NeuronJ.

### 2.11. Colorimetric MTT Assay

The metabolic viability of cultured cells was measured by the colorimetric MTT (3-(4,5-dimethylthiazol-2-yl)-2,5-diphenyltetrazolium bromide) (Thermo Fisher Scientific). MTT solution (0.5 mg/mL final concentration) was added to cultured cells and incubated for 4 h and 1.5 h for primary cells and BV-2 cells, respectively. Following incubation, the cells were lysed and MTT-formazan was solubilized in DMSO with an orbital shaker for 5 min. The optical density of each sample was determined using SpectraMax*®* M5 Microplate Reader (Molecular Devices, Sunnyvale, CA, USA) at 570 nm.

### 2.12. LDH Release Assay

Cell injury was quantitatively assessed by measuring lactate dehydrogenase (LDH) in the media. Briefly, cell-free culture medium was collected, and the amount of LDH released into the media was measured by using the Cytotoxicity Detection Kit (Roche) according to the manufacturer's protocol. The results were read on a SpectraMax M5 Microplate Reader (Molecular Devices). The amount of the red formazan product formed is proportional to the number of lysed cells. Percent cell injury was determined as experimental LDH release/total LDH release after lysis buffer-induced death × 100.

### 2.13. Enzyme-Linked Immunosorbent Assays (ELISA)

Following the stimulation of the cells with LPS, A*β*, and IFN-*γ*, supernatants were collected and assayed using commercial ELISA kits according to the manufacturer's instructions. Mouse IL-6, TNF-*α*, and CCL-2 kits were all from eBioscience (San Diego, CA, USA), and mouse CCL-5 ELISA kit was purchased from R&D (Minneapolis, MN, USA).

### 2.14. Measurement of NO Production

NO concentrations were determined by measurement of nitrite released into culture supernatants using the Griess Reagent System (Promega, Madison, WI, USA). Fifty microliter cell culture supernatants were incubated with 100 *μ*L Griess reagent in each well of a translucent 96-well plate. After incubation for 10 min at room temperature, absorbance was measured at 550 nm on a SpectraMax M5 Microplate Reader (Molecular Devices). Nitrite concentrations were calculated on the basis of a sodium nitrite reference curve.

### 2.15. Immunoblotting Analysis for BV-2 Cells

Total cell lysates were extracted with RIPA lysis buffer supplemented with protease inhibitor cocktail, phosphatase inhibitor cocktail, and 1 mM PMSF (all from Sigma-Aldrich). Protein levels were determined using the Bradford reagent (Sigma-Aldrich). Equal amounts of protein samples (20–80 *μ*g) were separated on a NuPAGE 4–12% Bis-Tris gel (Thermo Fisher Scientific) and transferred to a polyvinylidene difluoride membrane (Amersham Biosciences, Piscataway, NJ, USA) by iBlot® Dry Blotting System (Thermo Fisher Scientific). The membranes were blotted with primary antibodies against phospho-NF-*κ*B p65 (Ser536, 1 : 1000), NF-*κ*B p65 (1 : 1000), phospho-ERK1/2 (Thr202/Tyr204, 1 : 1000), ERK1/2 (1 : 1000), and *β*-actin (1 : 10,000) (all from Cell Signaling Technology), followed by a secondary antibody conjugated with HRP (Santa Cruz, Dallas, Texas, USA). Membranes were developed using Immobilon Kit (Merck Millipore, Darmstadt, Germany) according to the manufacturer's instructions and visualized by BioSpectrum® Imaging System (UVP; Cambridge, UK). Digital images were quantified by densitometry using AlphaImager 2200 v4.0 (Alpha Innotech Corporation, CA, USA) and adjusted for protein loading by normalizing to *β*-actin.

### 2.16. Quantitative Real-Time PCR Analysis

Messenger RNA (mRNA) was extracted from cells using the GeneJET RNA Purification Kit (Thermo Fisher Scientific) according to the manufacturer's instructions. First-strand cDNA was synthesized with the use of 1 *μ*g of total RNA (RevertAid™ First Strand cDNA Synthesis Kit). Primers were designed using the Roche Universal ProbeLibrary (UPL) (https://lifescience.roche.com/global_en.html) and were subjected to BLAST analysis to evaluate their specificity. The primers used in this study are listed in Table 1 and were assessed experimentally by PCR and agarose gel electrophoresis before quantitative real-time PCR (qPCR) experiments were conducted. The specificity of the primers was confirmed by the presence of a single peak in the melt curve generated for the genes. Amplification efficiencies ranged from 94.1% to 104.3%. qPCR was performed using Maxima SYBR Green qPCR Master Mix and run on a StepOnePlus Real-Time PCR System (all from Thermo Fisher Scientific). Quantification of RNA was determined by comparing the threshold cycle number of each gene to the GAPDH gene using the 2−^△△Ct^ method.

### 2.17. Statistical Analysis

All quantitative measures are presented as mean ± standard error of the mean or SEM. Statistical differences between the treatment groups were analyzed by one-way ANOVA followed by post hoc Dunnett's multiple comparison test. Alternatively, Student's *t*-test was used to compare between two samples as indicated in figure legends. *P* < 0.05 was considered statistically significant.

## 3. Results and Discussion

### 3.1. RC Protects Neurons against Fibrillar A*β* Toxicity

**Figure 1:**
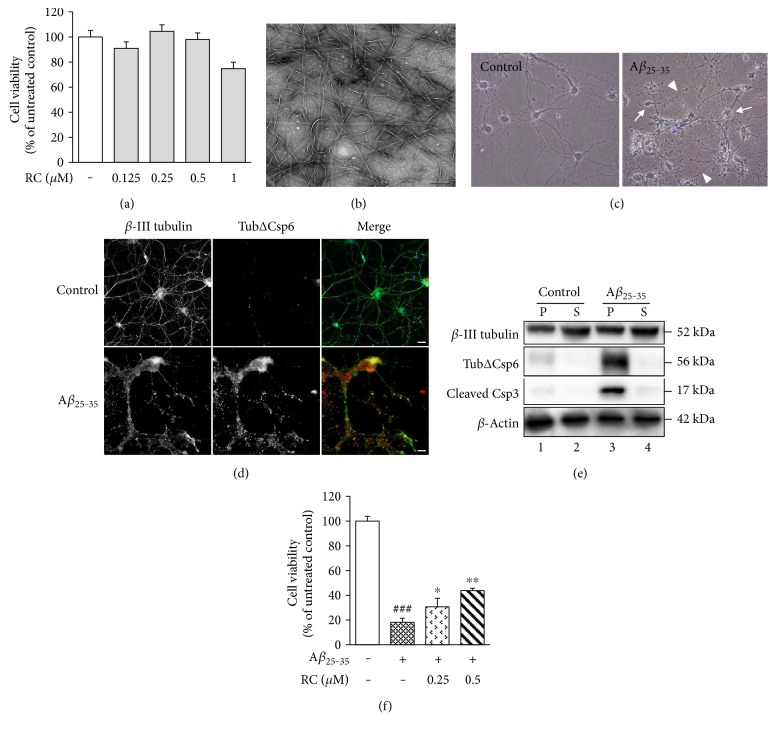
Protective effect of RC on A*β*_25–35_-induced cytotoxicity. (a) Toxicity of RC on primary rat hippocampal neurons was determined using the MTT assay after 24 h of incubation with RC at the indicated concentrations. Data are expressed as mean ± SEM (*n* = 3). Statistical analysis was performed by Student's *t*-test, and no significant difference was noted. (b) A representative image of A*β*_25–35_ after incubation at 37°C for 7 days. A*β*_25–35_ formed fibrillar morphology with ribbon-like structure. Scale bar, 200 nm. (c) Rat hippocampal neurons were treated with 0.1% DMSO or 25 *μ*M A*β*_25–35_ fibrils for 24 h and observed by phase-contrast microscopy. Note that cells treated with A*β*_25–35_ caused cell shrinkage (arrows) and neurite breakdown (arrowheads). (d) Cells with the above treatments were fixed and visualized by immunofluorescence microscope using anti-*β*-III tubulin antibody (green channel) costained with antitubulinΔCsp6 antibody that specifically recognized caspase-6-cleaved tubulin (red channel). Nuclei were revealed by staining with DAPI. Merged images show the superimposition of both green and red signals, with overlapping areas appearing yellow. A*β*_25–35_ treatment induced caspase-mediated cleavage of tubulin, indicative of apoptosis. (e) Rat hippocampal neurons were treated with 0.1% DMSO or 25 *μ*M A*β*_25–35_ for 24 h. The supernatant (S) and pellet (P) fractions were prepared from these cultures and analyzed by immunoblotting using the indicated antibodies. Caspase-6-cleaved tubulin (tubulinΔCsp6) and caspase-3-cleaved fragment (cleaved Csp3) indicate apoptosis. (f) RC reverses A*β*-induced cytotoxicity. Rat hippocampal neurons were pretreated with RC (0.25 and 0.5 *μ*M) for 1 h, followed by 24-hour incubation with 25 *μ*M A*β*_25–35_. Cell viability was measured by the MTT assay. Data are represented as mean ± SEM (*n* = 3). Statistical analysis was performed using one-way ANOVA with post hoc Dunnett's test. ^###^*P* < 0.001 versus control; ^∗^*P* < 0.05 and ^∗∗^*P* < 0.01 versus A*β*_25–35_.

As an initial assessment, we investigated whether the RC was directly toxic to neurons by incubating rat hippocampal neurons with concentrations of 0.125–1 *μ*M RC for 24 h. MTT assay revealed no significant toxicity in these cultures with 0.125, 0.25, or 0.5 *μ*M RC (Figure 1(a)). Based on these results, we chose to use concentrations of RC at or below 0.5 *μ*M for our studies unless otherwise stated.

Accumulating evidence suggests that neurotoxicity of aggregated A*β* isoforms can trigger neurodegeneration in AD [46]. To examine whether RC promotes neuronal survival after A*β* damage, we treated rat hippocampal neurons with A*β*_25–35_ fibrils (Figure 1(b)) by its features mimic full length A*β* to cause neuronal shrinkage (Figure 1(c), arrows) and neurite breakdown (Figure 1(c), arrowheads) and apoptotic reactions as indicated by caspase-6-cleaved *α*-tubulin (Figure 1(d), red channel; Figure 1(e), lane 3) and caspase-3-cleaved fragment (Figure 1(e), lane 3). Incubation of neurons with 25 *μ*M A*β*_25–35_ for 24 h greatly decreased cell viability compared to control neurons (Figure 1(f), *P* < 0.001). However, pretreatment of RC significantly reversed A*β*_25–35_-induced neuronal toxicity at concentrations of 0.25 *μ*M (Figure 1(f), *P* < 0.05) and 0.5 *μ*M of RC (Figure 1(f), *P* < 0.01).

### 3.2. RC Promotes Neurite Outgrowth in A*β*-Stimulated Neurons

**Figure 2:**
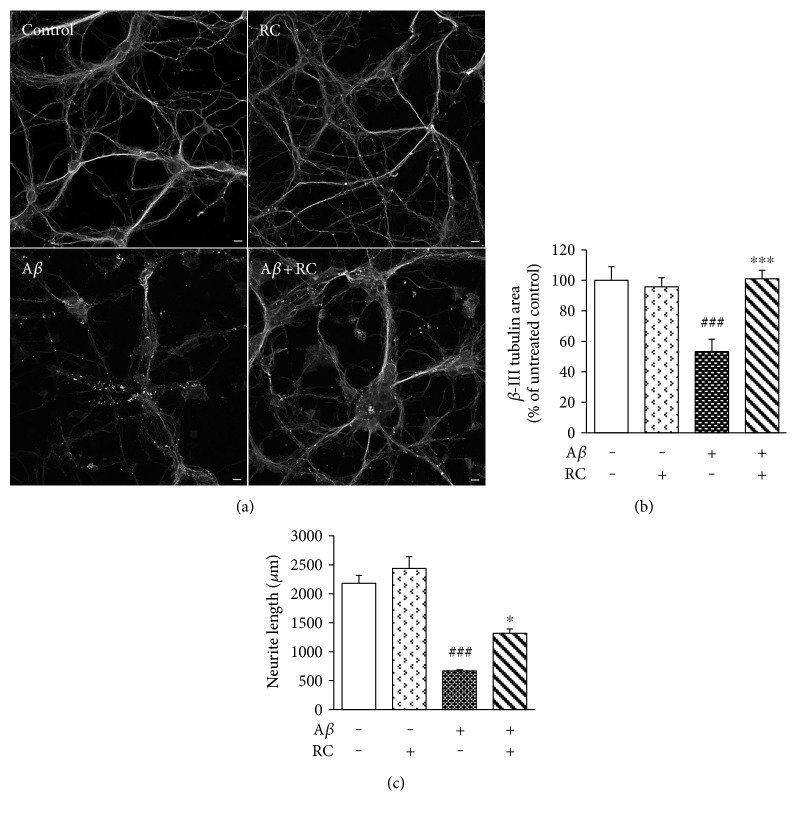
RC enhances neurite outgrowth in A*β*-damaged neurons. Rat hippocampal neurons were stimulated with 5 *μ*M A*β* for 24 h after 1 h of exposure to 0.5 *μ*M RC. Neuronal integrity was assessed by *β*-III tubulin immunocytochemistry (a) and image analysis (b). Note that all images were acquired at the same magnification. Scale bar, 10 *μ*m. (c) Total length of axons and minor neurites were quantified by NeuronJ software. Data are represented as mean ± SEM (*n* = 3). One-way ANOVA (post hoc Dunnett's test) was used to determine the significance of the data. ^###^*P* < 0.001 versus control; ^∗^*P* < 0.05 and ^∗∗∗^*P* < 0.001 versus A*β*.

The toxic effects of A*β* during the early progression of AD include pathological changes of neuronal morphologies, such as synapse loss, neurite dystrophy, and dendritic simplification [47]. Given that RC shows reversal activity on A*β* toxicity, it is tempting to speculate whether RC has beneficial effects on neurite integrity after A*β* injuries. As illustrated in our microphotographs, control cells exhibited *β*-III tubulin expression and intact cell bodies with elaborate networks of neurites in rat hippocampal neurons (Figure 2(a)). Although neuronal integrity (measured as total *β*-III tubulin area normalized to percentage of untreated neurons) was significantly decreased by A*β* by an average of 50% after 24 h (Figure 2(b), *P* < 0.001), RC restored neuronal integrity in A*β*-treated hippocampal neurons to levels of untreated neurons (Figure 2(b), *P* < 0.001). The neuroprotection of RC against A*β* damage was also validated by measuring neurite outgrowth. While A*β* significantly diminished neurite length in rat neurons (Figure 2(c), *P* < 0.001), enhanced neurite outgrowth was observed in neurons incubated with RC (0.5 *μ*M) when compared to A*β*-treated neurons (Figure 2(c), *P* < 0.05). Together, these results suggest that RC exerts protective potential on A*β*_25–35_-stimulated toxicity and neurite breakdown. We report for the first time that RC is a potent neuroprotective molecule against A*β* fibrils, which are considered to be one of the principal toxic forms of A*β* [48]. A*β* has been previously demonstrated to be able to prime microglia, whose activation is principally involved in the maintenance and progression of neuroinflammation [49]. Thus, it is likely that inhibition of the neurotoxic effects seen here is mediated through suppression of neuroinflammation.

### 3.3. RC Suppresses LPS-Induced Inflammatory Responses in Rat Glial Cultures

**Figure 3:**
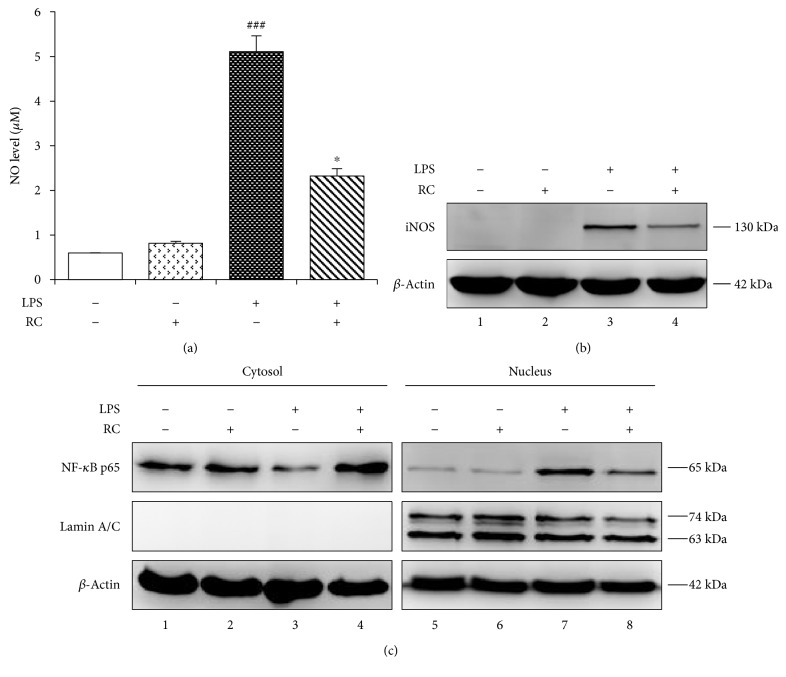
RC attenuates LPS-induced inflammatory responses in glia. Primary rat glial cultures were pretreated with 0.5 *μ*M RC for 1 h followed by incubation with 2 *μ*g/mL LPS for 24 h. Cell culture medium was used for NO production, and cell lysates were detected for iNOS expression or NF-*κ*B translocation. (a) NO levels were determined by Griess reagent. All values are expressed as mean ± SEM (*n* = 3). Data were analyzed using one-way ANOVA for with post hoc Dunnett's test. ^###^*P* < 0.001 versus control; ^∗^*P* < 0.05 versus LPS. (b) Representative images of iNOS expression in whole cell lysates resolved by immunoblotting (*n* = 2). *β*-Actin was used as a loading control. (c) NF-*κ*B levels in the cytosolic and nuclear fractions were evaluated by using immunoblot analysis. Shown are the representative blots (*n* = 2). Loading controls were *β*-actin (cytosolic) and lamin A/C (nuclear).

Emerging evidence supports the concept that persistent neuroinflammation is a major driver of neurodegeneration [1]. Indeed, the release of large amounts of NO generated by iNOS from activated microglia and astrocyte can react with ROS and produce reactive nitrogen species (RNS) that cause neurodegeneration of neurons [50]. To investigate the ability of RC to regulate neuroinflammation, we first treated mixed glial cultures (astrocytes and microglia) with LPS, the well-known potent inflammatory agent [51]. As NO has long been considered part of the neurotoxic insult caused by neuroinflammation, we determined the effect of RC on the LPS-stimulated production of NO in rat glial cells. Compared with unstimulated cells, NO secretion was markedly increased upon treatment with 2 *μ*g/mL LPS (Figure 3(a), *P* < 0.001). Nevertheless, RC (0.5 *μ*M) exposure for 30 min prior to LPS stimulation significantly inhibited NO production in mixed glia (Figure 3(a), *P* < 0.05). No effect on cell viability was observed in glia after incubation with RC or/and LPS (data not shown). Next, the effect of RC on LPS-induced iNOS expression was tested by immunoblotting. As shown in Figure 3(b), we found RC pretreatment resulted in an approximate 40% decrease in iNOS expression in LPS-stimulated glial cells (lane 4). Since NF-*κ*B is critical in LPS-induced inflammatory response [52], we further investigated whether the effects of RC in mixed glia were mediated via NF-*κ*B signaling pathway. As shown in Figure 3(c), rat glial cells in response to LPS were observed a remarkable increase in nuclear translocation of NF-*κ*B p65 subunit (lane 7), which was diminished by RC pretreatment by nearly 2.5-fold (lane 8). Overall, these data show that RC substantially inhibits NF-*κ*B-dependent inflammatory responses of glia.

### 3.4. RC Inhibits Microglial Activation in Rat Microglia

**Figure 4:**
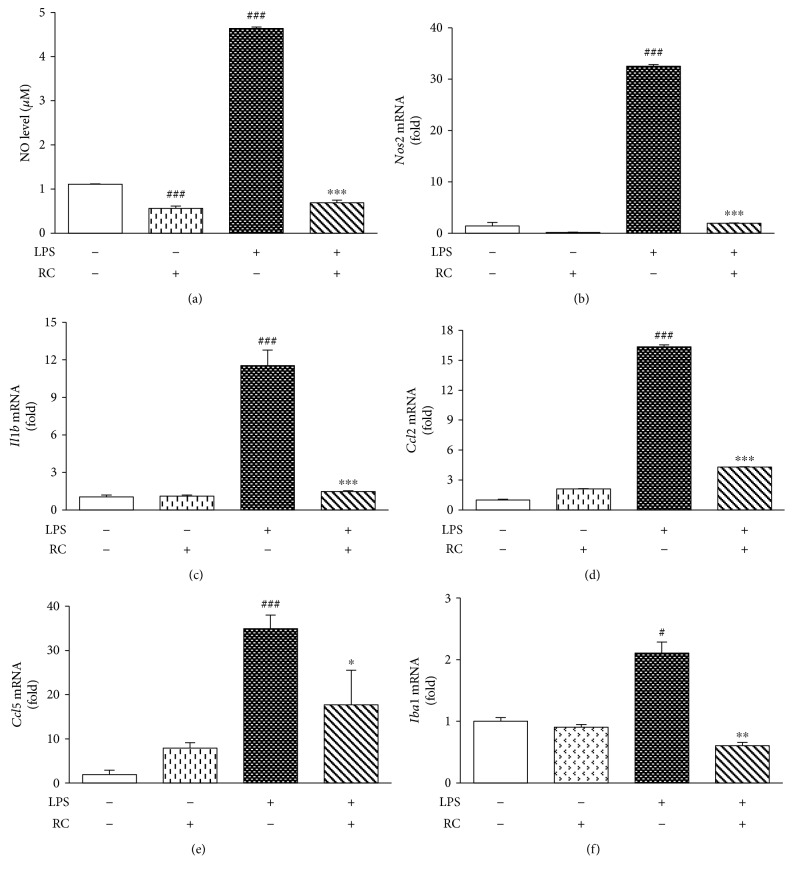
RC suppresses LPS-evoked microglial activation. Primary rat microglia were pretreated with 0.5 *μ*M RC for 1 h prior to the addition of 2 *μ*g/mL LPS for 24 h. (a) Cell culture supernatant was assessed for NO production by Griess reagent. (b–f) Quantitative PCR was used to assess transcript levels of *Nos2*, *Il1b*, *Ccl2*, *Ccl5*, and *Iba1*. All values are expressed as mean ± SEM (*n* = 3). Data were analyzed using one-way ANOVA with post hoc Dunnett's test. ^#^*P* < 0.05 and ^###^*P* < 0.001 versus control; ^∗^*P* < 0.05, ^∗∗^*P* < 0.01, and ^∗∗∗^*P* < 0.001 versus LPS.

Only recently has neuroinflammation emerged as Alzheimer's disease pathology, which involves microglial activation [53]. LPS is widely accepted as a means of inducing microglial activation in models of inflammatory neurodegeneration [54–56]. Indeed, it has been demonstrated that LPS-induced neuroinflammation in the brain of APPswe Tg mice increases intracellular accumulation of amyloid precursor protein (APP) and A*β*1–40/1–42 [57]. Also, systemic challenge with LPS on mouse brain stimulates microglial activation, neurodegeneration, and cognitive decline [58, 59]. Furthermore, LPS-stimulated microglia or monocytes caused neuronal cells to increase their expression of APP and release of its *β*-secretase-cleaved products (sAPP*β*s) as well as A*β* oligomers [60, 61]. Therefore, we utilized rat microglia stimulated by LPS to examine the effects of RC on microglia-mediated microglial activation. Upon LPS stimulation, NO production was remarkably elevated in rat microglia compared to control cells (Figure 4(a), *P* < 0.001). This increase in NO levels was dramatically reverted in cells pretreated with RC (Figure 4(a), *P* < 0.001). No effect on cell viability was detected in rat microglia after treatment with RC or/and LPS (data not shown). Furthermore, qPCR data revealed that LPS-treated microglia exhibited increased expression of inflammatory mediators such as *Nos2*, *Il1b*, *Ccl2*, and *Ccl5*, whereas RC significantly attenuated mRNA levels of these factors (Figures 4(b), 4(c), 4(d), and 4(e)).

More recently, Norden et al. have revealed that the expression levels of microglial marker Iba-1 (ionized calcium-binding adaptor molecule-1) was elevated in mice challenged with LPS [62]. Similarly, in the present study, LPS-induced increase of *Iba1* gene expression was also observed in rat microglia (Figure 4(f), *P* < 0.05). However, the effect was remarkably suppressed by RC treatment (Figure 4(f), *P* < 0.01). This finding is consistent with the well-known fact that Iba-1 is overexpressed in activated microglia during the neuroinflammatory response [63]. Altogether, our data provided the evidence that in activated microglia, RC exerts beneficial effects by targeting iNOS in nitrite production as well as by modulating cytokine expression levels.

### 3.5. RC Decreases IL-6 and TNF-*α* Production in LPS-Activated BV-2 Microglia

**Figure 5:**
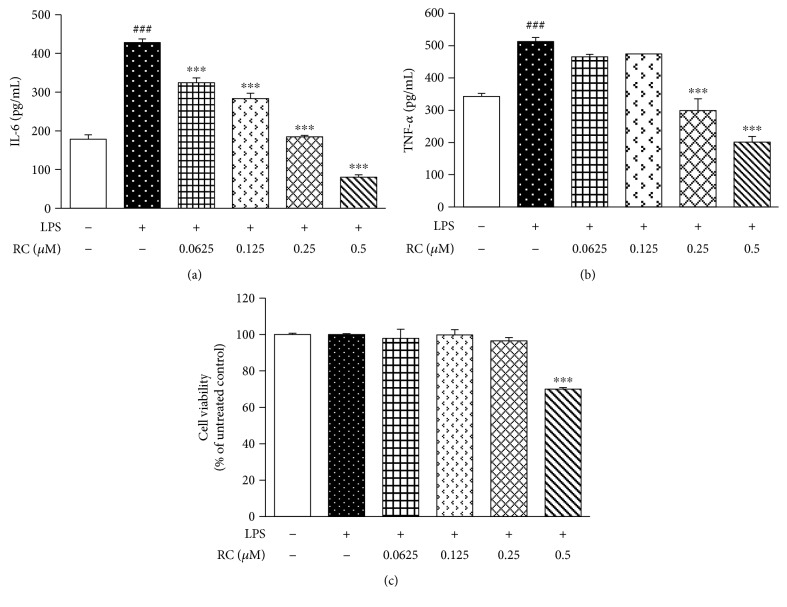
RC inhibits IL-6 and TNF-*α* secretion in LPS-activated microglia. BV-2 cells were treated with LPS (100 ng/mL) for 24 h following 1 h exposure to RC (0.625–0.5 *μ*M). Cell supernatants were collected for IL-6 (a) and TNF-*α* (b) detection by ELISA. Data are represented as mean ± SEM (*n* = 3). The significance of the data were analyzed by one-way ANOVA with post hoc Dunnett's test. ^###^*P* < 0.001 versus control; ^∗∗∗^*P* < 0.001 versus LPS. (c) Cell viability was assessed by the MTT assay. Statistical analysis was performed by Student's *t*-test. ^∗∗∗^*P* < 0.001 versus LPS.

To investigate the neuroinflammation aspect further, the BV-2 mouse microglial cell line was used as an alternative model system for primary microglial culture [64]. The activated BV-2 model is frequently used to evaluate the efficacy and potency of immunomodulating agents against endotoxic production of NO, IL-6, TNF-*α*, IL-1*β*, CCL-2, or NADPH oxidase, contributors to CNS neurodegenerative injury [65, 66]. Consequently, we investigated whether RC would prevent the production of cytokines following LPS activation of BV-2 microglia. ELISA analysis of BV-2 cells demonstrated that RC attenuated LPS-induced production of IL-6 and TNF-*α* (Figures 5(a) and 5(b)), both of which are known to promote autocrine signaling in microglia. The reduction of IL-6 and TNF-*α* by RC was not resulted from cytotoxicity, as confirmed by the MTT assay (Figure 5(c)). Given that both IL-6 and TNF-*α* can compromise neuron survival [21], these findings raise the possibility that RC may be able to protect neuronal populations that display increased vulnerability to inflammation-induced apoptotic death, by limiting the production of these neurotoxic factors by chronically activated microglia.

### 3.6. RC Reduces IL-6 and TNF-*α* Secretion from A*β*- and IFN-*γ*-Activated BV-2 Microglia

**Figure 6:**
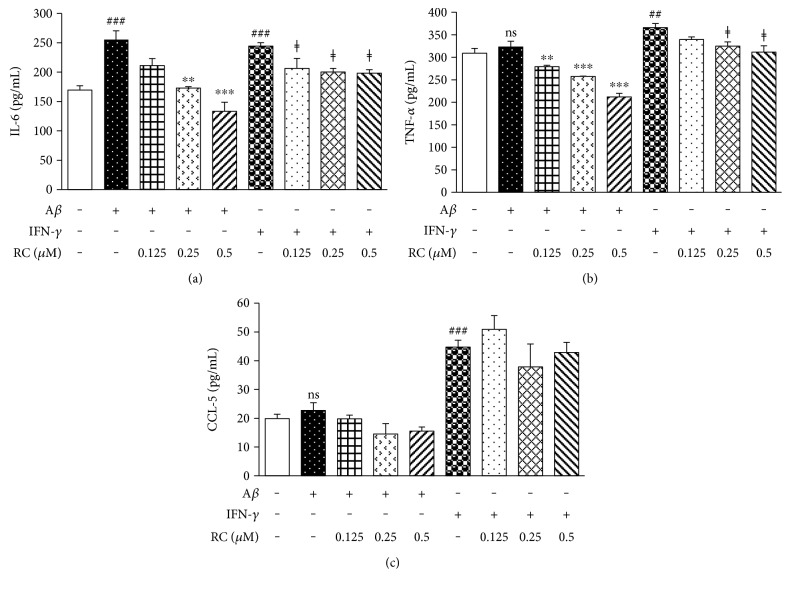
RC decreases proinflammatory cytokine production in A*β*- and IFN-*γ*-stimulated microglia. BV-2 cells were exposed to A*β* (20 *μ*M) or IFN-*γ* (100 pg/mL) for 48 h following the pretreatment of RC (0.125–0.5 *μ*M) for 1 h. Cell supernatants were collected for IL-6 (a), TNF-*α* (b), and CCL-5 (c) measurements by ELISA. All values are expressed as mean ± SEM (*n* = 3). Data were analyzed using one-way ANOVA with post hoc Dunnett's test. ^##^*P* < 0.01, ^###^*P* < 0.001, and ns (not significant) versus control; ^∗∗^*P* < 0.01 and ^∗∗∗^*P* < 0.001 versus A*β*; ^ǂ^*P* < 0.05 versus IFN-*γ*.

Numerous studies suggest that cytokine-induced neuroinflammation contributes to the clinical progression of AD [67, 68]. While it is difficult to ascertain the extent of cytokine secretion that occurs within the brain directly, the addition of fibrillar forms of A*β*_25–35_ or IFN-*γ* stimulates the secretion of cytokines from cultured macrophages and microglia [69, 70]. Thus, we investigated the effects of RC on A*β*- and IFN-*γ*-stimulated BV-2 microglia. As shown in Figure 6(a), IL-6 was elevated in microglia exposed to 20 *μ*M A*β* or 100 pg/mL IFN-*γ* for 48 h (*P* < 0.001), whereas pretreatment with RC (0.125–0.5 *μ*M) significantly prevented IL-6 production. The addition of A*β* caused slight but not significant TNF-*α* production over that of control cells, which is similar to the results found by Couch and colleagues [71]. However, RC treatment significantly reduced the secretion of TNF-*α* from BV-2 cells incubated with A*β* or IFN-*γ* (Figure 6(b)) without observed cytotoxicity (data not shown).

**Figure 7:**
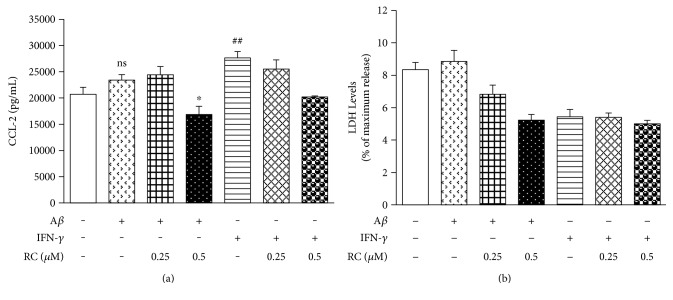
RC diminishes CCL-2 secretion in A*β*- and IFN-*γ*-stimulated microglia. BV-2 cells were stimulated with A*β* (20 *μ*M) or IFN-*γ* (100 pg/mL) for 24 h after 1 h incubation with RC (0.25 and 0.5 *μ*M). (a) Cell supernatants were collected for CCL-2 measurements by ELISA. All values are expressed as mean ± SEM (*n* = 3). Data were analyzed using one-way ANOVA with post hoc Dunnett's test. ^##^*P* < 0.01 and ns (not significant) versus control; ^∗^*P* < 0.05 versus A*β*. (b) Cytotoxicity was measured by LDH release assay. No significant cell death was observed.

Besides chemoattractant function to recruit monocytes and macrophages into the brain, CCL-5 and CCL-2 have a modulatory effect on activated microglia [72, 73]. Hence, the effects of RC on CCL-5 and CCL-2 secretion were also tested in BV-2 microglia treated with A*β* or IFN-*γ* for 24 h. We found that RC treatment showed a tendency to suppress CCL-5 production in BV-2 microglia exposed to A*β* but not to IFN-*γ* (Figure 6(c)). Pretreatment of BV-2 cells with 0.5 *μ*M RC reduced the levels of CCL-2 induced by A*β* or IFN-*γ* (Figure 7(a)). This inhibition of cytokines was not due to cytotoxicity, as determined by LDH release assay (Figure 7(b)). Altogether, these data suggest that RC has an inhibitory effect on the secretion of inflammatory mediators including IL-6, TNF-*α*, and CCL-2 in BV-2 cells. In line with our findings, Chang et al. [34] reported that the RC used in their study inhibited IL-1*β*, IL-6, and TNF-*α* mRNA in the rat cerebral spinal fluid samples with subarachnoid hemorrhage, while suppression of LPS-evoked TNF-*α* secretion from RAW264.7 mouse macrophages with RC was revealed by Tewtrakul et al. [33] Since these cytokines are all reported NF-*κ*B-driven genes, we further investigated the pathways modified by RC.

### 3.7. RC Abrogates NF-*κ*B and ERK Activation in A*β*- or IFN-*γ*-Stimulated Microglia

**Figure 8:**
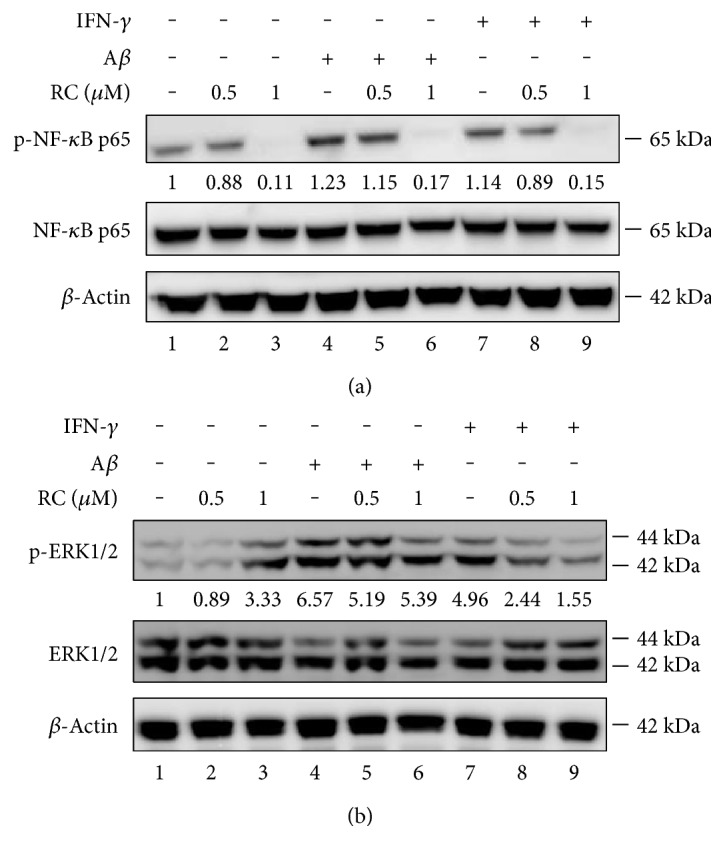
RC abrogates A*β*- and IFN-*γ*-induced NF-*κ*B and ERK activation. BV-2 cells were stimulated with 20 *μ*M A*β* or 100 pg/mL IFN-*γ* for 3 h following the 4 h pretreatment of 0.5 and 1 *μ*M RC. Whole cell lysates were collected and subject to immunoblotting for the indicated proteins. Representative images were shown for p-NF-*κ*B and NF-*κ*B (a) and p-ERK and ERK (b) (*n* = 2). *β*-Actin was used as an internal control. Quantification of blots was performed by using AlphaImager 2200 v4.0 and the fold changes of p-NF-*κ*B to NF-*κ*B (a) and p-ERK to ERK (b) are presented.

The transcription factor NF-*κ*B is a critical regulator of immune and inflammatory responses [52]. Considerable evidence suggests that the activation of NF-*κ*B in the CNS triggers multicellular responses and gene transactivation intricately associated with the initiation and progression of neurodegenerative diseases. Various endogenous and exogenous stimuli activate NF-*κ*B-enhancing transactivation of inflammatory molecules and production of free radicals in glial cells [74]. In order to establish whether RC reduces inflammatory cytokine production via the NF-*κ*B pathway, we tested the effect of RC on the p65 subunit of NF-*κ*B. BV-2 microglia were pretreated with RC for 4 h prior to the incubation with 20 *μ*M A*β* or 100 pg/mL IFN-*γ* for 3 h. As shown in Figure 8(a), A*β* or IFN-*γ* led to higher phosphorylation of the p65 subunit (lanes 4 and 7) compared to the control counterpart (lane 1). On the other hand, RC at 0.5 and 1 *μ*M reduced A*β*- or IFN-*γ*-activated NF-*κ*B p65 phosphorylation (Figure 8(a), lanes 5, 6, 8, and 9).

Previous studies showed that ERK regulates NF-*κ*B-dependent transcription and plays a role in neuroinflammation and AD [75]. To determine possible effects of RC on the activities of ERK, we pretreated BV-2 microglia with RC (0.5 and 1 *μ*M) for 4 h, followed by 20 *μ*M A*β* or 100 pg/mL IFN-*γ* stimulation for 3 h. Our results revealed that phosphorylation of ERK was remarkably elevated in A*β*- or IFN-*γ*-activated BV-2 cells (Figure 8(b), lanes 4 and 7), whereas RC reduced ERK activation (Figure 8(b), lanes 5, 6, 8, and 9). Taken together, our findings suggest that RC targets the NF-*κ*B and ERK signaling pathways and subsequently abrogates the expression of IL-6, TNF-*α*, and CCL-2 in BV-2 microglia. In line with the potential therapeutic implications in the compounds which mitigate NF-*κ*B [76] or ERK [77] signaling, it is hypothesized that the microglia-mediated neurotoxicity by LPS, A*β*, and IFN-*γ* is probably ameliorated by RC through suppression of NF-*κ*B- and ERK-mediated pathways.

### 3.8. RC Reduces the Toxic Effects of Conditioned Media from Microglia Treated with A*β* on Hippocampal Neurons

**Figure 9:**
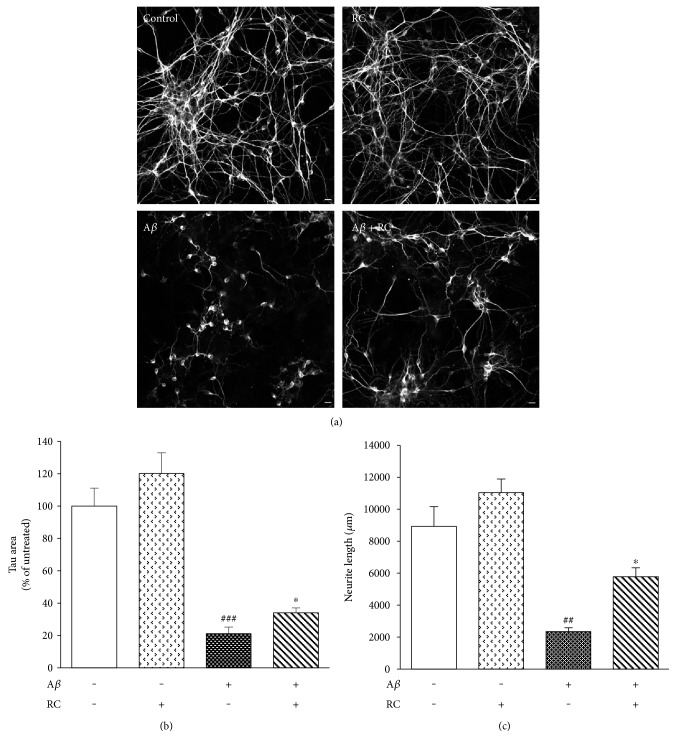
RC reduces damage to primary neurons treated with conditioned media from A*β*-stimulated microglia. Rat microglia were untreated or pretreated with RC (0.5 *μ*M) for 1 h followed by 24 h exposure to A*β* (5 *μ*M). The culture media were centrifuged, filtered, and used as conditioned media. Rat hippocampal neurons at 14 days in culture were treated with 50% conditioned media from microglia for 24 h. (a) Neuronal integrity was assessed by immunostaining with anti-Tau antibody that specifically labeled axonal processes. Note that addition of the conditioned media from A*β*-stimulated microglia induced neuronal damage, and the presence of RC partially reversed this effect. The conditioned media from RC-treated microglia had no detectable effect on the morphological appearance of neurons when compared to those grown in control medium. (b) Tau immunoreactivity was quantified with ImageJ. Scale bar, 20 *μ*m. (c) Total lengths of axons were measured by NeuronJ. Data are represented as mean ± SEM (*n* = 3). One-way ANOVA (post hoc Dunnett's test) was used to determine the significance of the data. ^##^*P* < 0.01 and ^###^*P* < 0.001 versus control; ^∗^*P* < 0.05 versus A*β*.

Previous reports have indicated that activated microglia are crucial mediators of A*β*-induced neurotoxicity through the secretion of toxic factors including ROS, NO, and cytokines during neuroinflammation [78, 79]. Also, A*β*_25–35_ fibrils exert a cytotoxic effect on neurons and stimulate microglia to produce neurotoxins in rat microglia [23, 80, 81]. Accordingly, we evaluated the effect of RC in the generation of putatively toxic conditioned media derived from rat microglia treated with A*β*_25–35_ fibrils. As shown in Figure 9, conditioned media obtained from microglia exposed to 5 *μ*M A*β* for 24 h reduced Tau immunoreactivity by 80% in hippocampal neurons (Figures 9(a) and 9(b), *P* < 0.001) as well as decreased axon length by 75% compared to control (Figure 9(c), *P* < 0.01). In contrast, conditioned media obtained from microglia pretreated with 0.5 *μ*M RC followed by 24 h A*β* incubation restored neuronal integrity as demonstrated by increased Tau immunoreactivity and axon outgrowth compared to A*β* treatment (Figures 9(a), 9(b), and 9(c)). These observations indicate that RC is able to ameliorate microglia-mediated neurotoxicity. Along with our unpublished data showing that A*β*_25–35_ increases the expression of inflammatory mediators such as IL-6, TNF-*α*, IL-1*β*, CCL-2, and CCL-5 in rat hippocampal neuron culture, it is hypothesized that RC may reduce neuronal damage caused by microglial conditioned media through suppression of A*β*_25–35_-induced neuroinflammation. However, the exact mechanisms remain to be elucidated.

Increased production of A*β* fibrils can activate microglia via various receptors that cooperate in the recognition, internalization, and clearance of A*β*, including CD14, CD36, CD47, *α*6*β*1 integrin, class A scavenger receptor, receptor for advanced glycosylation end products (RAGE), and toll-like receptors (TLRs) [1]. Binding of A*β* to, for example, RAGE results in the production of inflammatory cytokines and reduces A*β* uptake in cultured microglia [82, 83] and in transgenic mice expressing human mutant APP [84]. Besides the production of inflammatory mediators upon binding of A*β* to various microglial receptors, it has been revealed that microglia could clear A*β* through receptor-mediated phagocytosis and degradation *in vitro*. Indeed, microglia possess the machinery to degrade the pathogenically more relevant A*β* species via extracellular proteases such as neprilysin, insulin-degrading enzyme (IDE), and matrix metalloproteinases [85]. In consideration of the function of RC in suppressing receptor activator of nuclear factor-*κ*B ligand- (RANKL-) stimulated osteoclastogenesis [35] and inhibiting ATP-binding cassette transporters (ABC transporters) in breast cancer cells [86], it is tempting to speculate that RC deters A*β* neurotoxicity probably through microglia-mediated clearance of A*β* or inflammatory responses via acting on receptors, membrane proteins, or degradation enzymes, although their precise mechanisms of interaction have yet to be defined.

## 4. Conclusions

**Figure 10:**
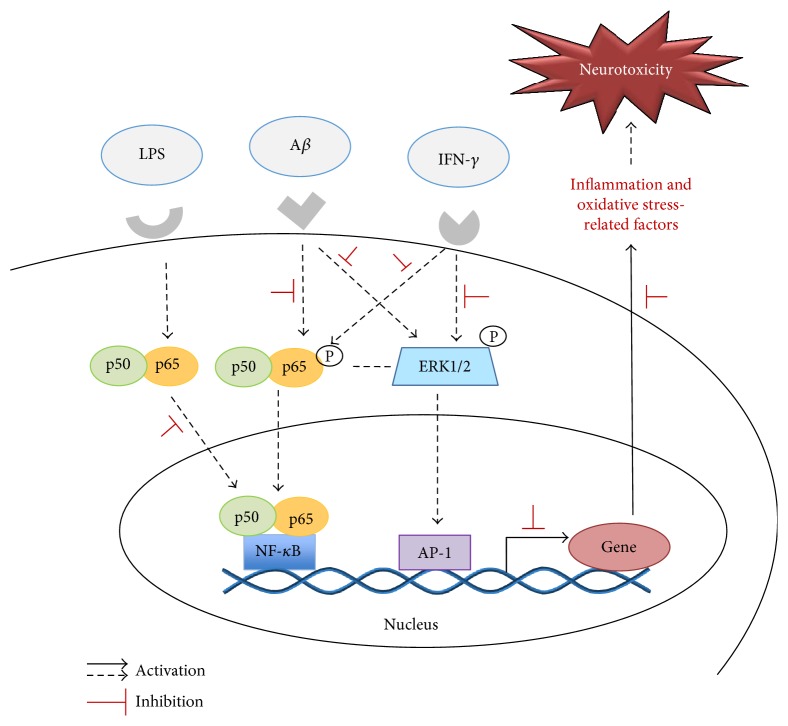
Schematic diagram showing the neuroprotective effect of RC via a mechanism involving NF-*κ*B- and ERK-mediated signaling. LPS, A*β*, and IFN-*γ* initiate the inflammation by activating NF-*κ*B and ERK pathways. The activation of NF-*κ*B and ERK regulates the gene expression of proinflammatory mediators and neurotoxic factors that ultimately cause neurotoxicity. However, RC inhibits the NF-*κ*B and ERK pathways to interfere with further inflammatory responses or neuronal damage.

Approaches to target A*β* via passive antibody or inhibition of *γ*-secretase therapy have not yet generated substantial clinical benefits, controlling the activation of microglia, and the resulting neuroinflammation has become a novel strategy for drug development for AD. Here, we report that RC is endowed with a neuroprotective role on processes that possibly influence microglial activation and neuroinflammation. Specifically, RC prevents cytotoxicity and enhances neurite outgrowth in A*β*-treated neurons. In response to LPS, RC inhibits NO secretion and inflammatory responses in primary rat glia. Likewise, RC regulates LPS-, A*β*- or IFN-*γ*-stimulated overproduction of inflammatory cytokines in BV-2 microglia. Of note, NF-*κ*B and ERK activation was suppressed in BV-2 cells exposed to A*β* and IFN-*γ*. Intriguingly, RC partially protects neurons from A*β*-stimulated microglial conditioned media-dependent toxicity. Overall, our results point to RC exerting potent antineuroinflammatory and neuroprotective effects plausibly via NF-*κ*B and ERK signaling pathways as depicted in Figure 10, which constitute a novel therapeutic measure to AD. To the best of our knowledge, this is the very first evidence for the anti-inflammatory effects of RC in microglia. In view of the most recently proposed antimicroglial agents targeting the proinflammatory phenotype would be beneficial in AD [87], further studies are required to determine the mechanisms through which RC regulates neurotoxicity linked to the microglial neuroinflammation and the exact functions of RC in the pathogenesis of AD. This could be of therapeutic relevance in the prospects of applying RC to slow or prevent AD-related pathology on microglia-based approach [8]. For these reasons, we are continuing to study and investigate these mechanistic questions in our laboratory. Accordingly, the potential of RC in preventing or treating AD will be examined in animal models in the future.

## Figures and Tables

**Table 1:** List of primers used.

Gene	Accession number	Forward (5′-3′)	Reverse (5′-3′)
*Il1b*	NM_031512.2	TGTGATGAAAGACGGCACAC	CTTCTTCTTTGGGTATTGTTTGG
*Nos2*	NM_012611.3	AGGTTGGAGGCCTTGTGTC	GCTTCAGAATGGGGAGCTG
*Ccl5*	NM_031116.3	CTCACCGTCATCCTCGTTG	GAGTGGTGTCCGAGCCATA
*Ccl2*	NM_031530.1	CGTGCTGTCTCAGCCAGAT	GGATCATCTTGCCAGTGAATG
*Iba1*	NM_017196.3	CCGAGGAGACGTTCAGTTACTC	TGGCTTCTGGTGTTCTTTGTT
*Gapdh*	NM_017008.4	ATGGCCTTCCGTGTTCCTAC	GCCTGCTTCACCACCTTCTT

## Abbreviations

A*β*: Amyloid-*β* peptide
ABC: ATP-binding cassette
AD: Alzheimer's disease
ANOVA: Analysis of variance
APP: amyloid precursor protein
BSA: Bovine serum albumin
CCD: Charge-coupled device
CCL-2: Chemokine (C-C motif) ligand 2/MCP-1
CCL-5: Chemokine (C-C motif) ligand 5/RANTES
CNS: Central nervous system
DAPI: 4′,6-Diamidino-2-phenylindole
DMSO: Dimethyl sulfoxide
ECL: Enhanced chemiluminescence
ELISA: Enzyme-linked immunosorbent assay
ERK: Extracellular signal-regulated kinases
GFAP: Glial fibrillary acidic protein
HBSS: Hank's balanced salt solution
HRP: Horseradish peroxidase
Iba-1: Ionized calcium-binding adapter molecule 1
IFN-*γ*: Interferon-*γ*
IL-1*β*: Interleukin-1*β*
IL-6: Interleukin-6
iNOS: Inducible nitric oxide synthase
LDH: Lactate dehydrogenase
LPS: Lipopolysaccharide
MEM: Modified Eagle's medium
MTT: 3-(4,5-Dimethylthiazol-2-yl)-2,5-diphenyltetrazolium bromide
NF-*κ*B: Nuclear factor kappa B
NGS: Normal goat serum
NO: Nitric oxide
PBS: Phosphate-buffered saline
PFA: Paraformaldehyde
PMSF: Phenylmethylsulfonyl fluoride
qPCR: Quantitative polymerase chain reaction
RAGE: Receptor for advanced glycosylation end products
RANKL: Receptor activator of nuclear factor-*κ*B ligand
RC: Rhinacanthin C
RIPA: Radioimmunoprecipitation assay
RPMI: Roswell Park Memorial Institute
RNS: Reactive nitrogen species
ROS: Reactive oxygen species
sAPP*β*s: *β*-Secretase-cleaved products
TBS: Tris-buffered saline
TLRs: Toll-like receptors
TNF-*α*: Tumor necrosis factor-*α*.
